# Tracking progress in suicide prevention in Indigenous communities: a challenge for public health surveillance in Canada

**DOI:** 10.1186/s12889-018-6224-9

**Published:** 2018-11-27

**Authors:** Nathaniel J. Pollock, Gwen K. Healey, Michael Jong, James E. Valcour, Shree Mulay

**Affiliations:** 1 Division of Community Health and Humanities, Faculty of Medicine, Memorial University, Prince Philip Drive, St. John’s, Newfoundland and Labrador A1B 3V6 Canada; 2 Labrador Institute of Memorial University, P.O. Box 490, Stn. B, 219 Hamilton River Road, Happy Valley-Goose Bay, Newfoundland and Labrador A0P 1E0 Canada; 3 Qaujigiartiit Health Research Centre, PO Box 11372, 764 Fred Coman Dr., Iqaluit, NT X0A 0H0 Canada; 4 Northern Ontario School of Medicine, Thunder Bay, ON Canada; 5 Labrador-Grenfell Regional Health Authority, Labrador Health Centre, Happy Valley-Goose Bay, Newfoundland and Labrador Canada; 6 Northern Family Medicine Program (NorFam), Discipline of Family Medicine, Faculty of Medicine, Memorial University, St. John’s, Newfoundland and Labrador A1B 3V6 Canada

**Keywords:** Suicide prevention, Self-harm, Epidemiology, Indigenous, Inuit, First Nations, Circumpolar, Administrative data, Data governance, Health disparities

## Abstract

Indigenous peoples in Canada experience disproportionate rates of suicide compared to non-Indigenous populations. Indigenous communities and organizations have designed local and regional approaches to prevention, and the federal government has developed a national suicide prevention framework. However, public health systems continue to face challenges in monitoring the population burden of suicide and suicidal behaviour. National health data systems lack Indigenous identifiers, do not capture data from some regions, and do not routinely engage Indigenous communities in data governance. These challenges hamper efforts to detect changes in population-level outcomes and assess the impact of suicide prevention activities. Consequently, this limits the ability to achieve public health prevention goals and reduce suicide rates and rate inequities.

This paper provides a critical analysis of the challenges related to suicide surveillance in Canada and assesses the strengths and limitations of existing data infrastructure for monitoring outcomes in Indigenous communities. To better understand these challenges, we discuss the policy context for suicide surveillance and examine the survey and administrative data sources that are commonly used in public health surveillance. We then review recent data on the epidemiology of suicide and suicidal behaviour among Indigenous populations, and identify challenges related to national surveillance.

To enhance capacity for suicide surveillance, we propose strategies to better track progress in Indigenous suicide prevention. Specifically, we recommend establishing an independent community and scientific governing council, integrating Indigenous identifiers into population health datasets, increasing geographic coverage, improving suicide data quality, comprehensiveness, and timeliness, and developing a platform for making suicide data accessible to all stakeholders. Overall, the strategies we propose can build on the strengths of the existing national suicide surveillance system by adopting a collaborative and inclusive governance model that recognizes the stake Indigenous communities have in suicide prevention.

## Background

Suicide is a leading cause of death among Indigenous peoples in Canada [1–5]. Although incidence rates vary by community and region [3, 6, 7], studies consistently show that Inuit, First Nations, and Métis have disproportionate rates of suicide compared to non-Indigenous populations [4–9]. Disparities in suicide mortality are so stark that suicide prevention has become a public health priority for many Indigenous communities and governments [9–14]. As a priority, governments and stakeholders have undertaken substantial efforts to understand the root causes of suicide [8, 9, 11, 13, 15] and to chart a path towards a “low-suicide reality” [16]. Despite all that is known about the causes and impacts of suicide, population-specific statistics are not widely available and often do not describe the full extent of the problem. The picture we have is only a partial one: of the approximately 4000 people who die by suicide in Canada each year, neither governments nor communities know how many were Indigenous.

The fourteen publicly-funded provincial, territorial, and federal health care systems in Canada are inconsistent in their approach to identifying Indigenous peoples in health information, and some provinces/territories, such as Newfoundland and Labrador, do not include ethnic identifiers in administrative or clinical data [17–19]. The omission of ethnic identifiers makes it difficult to measure changes in health status at the population level [20, 21]. Yet, a core responsibility of public health is to report on markers of population health such as mortality rates [22–24]. The need for Indigenous-specific population health data was underscored in several recent studies [25–27] and notably in 2015 by the Truth and Reconciliation Commission (TRC). The TRC’s Call to Action #19 states: “We call upon the federal government, in consultation with Aboriginal peoples, to establish measurable goals to identify and close the gaps in health outcomes between Aboriginal and non-Aboriginal communities, and to publish annual progress reports and assess long-term trends” [28]. The TRC pointed to key indicators for assessing health equity; suicide was among them [28]. This call gives Canadian public health organizations a specific, achievable mandate, and a role in reconciliation.

Progress on this particular TRC Call to Action has been slow because in addition to the lack of ethnic identifiers, several long-standing factors make it difficult to measure suicide. In 1995, the Royal Commission on Aboriginal Peoples identified challenges such as incomplete geographic coverage in some datasets and a lack of data on suicide attempts [15]. Data gaps still exist today, and national organizations including Inuit Tapiriit Kanatami, the Canadian Association for Suicide Prevention, and the Mental Health Commission of Canada and Indigenous governments have called for enhanced suicide surveillance capacity that includes accurate and Indigenous-specific data [9, 12, 29, 30]. Globally, the World Health Organization (WHO) recommended that all countries develop a national suicide prevention strategy that integrates a comprehensive suicide surveillance program with policy and interventions [31]. As recently as 2017, the Public Health Agency of Canada (PHAC) responded to the WHO’s call by developing the Canadian Suicide Surveillance Indicator Framework (CSSIF) [32], which was an essential step for suicide prevention in Canada. The inaugural CSSIF publication reported baseline incidence and prevalence rates for monitoring suicide and suicide-related outcomes [32]. But like many population health measurement strategies, the CSSIF does not include a mechanism for tracking outcomes among Indigenous peoples [32].

In recent years, Indigenous-focused suicide prevention programs have received major government investments but have operated without detailed data on the epidemiology of suicide. From 2005/06 to 2015/16, the federal government committed $108,000,000 to the National Aboriginal Youth Suicide Prevention Strategy [33, 34]. An evaluation described outputs of the strategy, such as the number and types of programs that were funded; however the evaluation explained that the lack of suicide surveillance data prevented both performance measurement and “a comprehensive assessment of the trends and achievement of long term outcomes (improved health status)” [33] (p.8). The absence of quantitative outcome data connected to the strategy meant that there was no clear picture of whether or not the NAYSPS had an impact, positive or negative, on suicide rates. At the most basic level, the lack of Indigenous-specific suicide data means that communities, health systems, and governments are unable to tell if suicides are being prevented.

Our objective for this paper is to offer a review of the challenges related to suicide surveillance in Canada and discuss strengths and limitations for monitoring outcomes related to suicide prevention among Indigenous peoples. We aim to address this by: (1) examining the policy context for suicide surveillance in public health; (2) describing the sources of population health data commonly used in suicide surveillance; (3) synthesizing recent data on the epidemiology of suicide among Indigenous populations; (4) identifying challenges related to Indigenous-specific suicide surveillance; and (5) proposing strategies to better track progress in Indigenous suicide prevention.

**Fig. 1:**
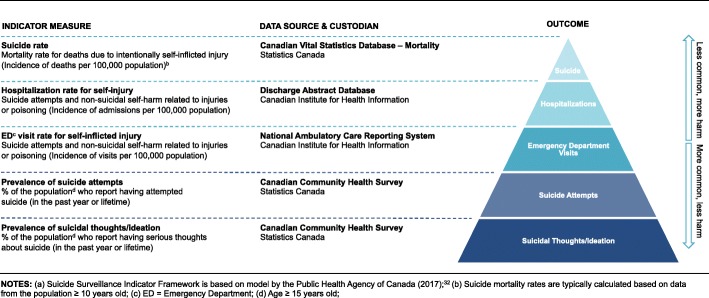
**a** Suicide Surveillance Indicator Framework. **b**: Iceberg Model of Suicide

The statistics reported in this paper are aligned with the national suicide surveillance framework (Fig. 1a) which includes the following indicators: suicide mortality, hospitalization due to self-injury, emergency department visits for self-injury, suicide attempts, and suicidal thoughts (also called suicidal ideation) [32]. PHAC uses “self-injury,” whereas other organizations and scholars choose terms such as “self-harm” and “suicide-related behaviours.” Broadly speaking, there is overlap in the definitions of these terms. They generally refer to a group of non-fatal outcomes that include suicide attempts, ‘parasuicide,’ other forms of intentional self-injury and self-poisoning, and behaviours where the intent is undetermined [31, 35]. Our use of terms in this paper reflects those used in the original data sources.

### The policy context for suicide surveillance

Public health surveillance is the systematic and ongoing process of collecting, analyzing, and interpreting data on the health of the population [23, 24]. Surveillance systems provide much needed information to decision-makers who are responsible for planning, funding, delivering, and evaluating interventions that fall under the umbrella of suicide prevention [31, 36, 37]. Health indicators are tools used in surveillance to measure various health outcomes and risk factors, and to establish reference points for tracking health status over time and in relation to health services, public health interventions, and social conditions [23, 24]. Public health surveillance systems are useful because they can help detect changes that occur among subgroups of people, uncover new risk and protective factors, identify and predict disease outbreaks, and mobilize interventions [23, 24, 31, 38]. To address our first objective, in this section we discuss the policy context for suicide surveillance in Canada. By highlighting some recent developments in public health approaches to suicide prevention, we aim to situate our subsequent analysis about the challenges related to surveillance.

In 2014, the World Health Organization released the landmark report, *Preventing Suicide: A Global Imperative* [31]. The report included a synthesis of the clinical and public health evidence in suicide prevention, and provided a framework for the creation of national suicide prevention strategies. One of WHO’s recommendations was that federal governments should develop a comprehensive suicide surveillance system as a part of a national strategy, and integrate surveillance with policy decisions and intervention evaluations. The recommendation was necessary because expertise and technical infrastructure for population health monitoring is varied and poor in many countries. Death registration, especially of suicide, is a complex process that crosses government sectors and is influenced by social norms and taboos [31, 39]. The WHO estimated that 112 of the 192 member states have low quality or non-existent death registration systems [31]. Despite having strong vital statistics systems, even high-income countries such as Canada face challenges related to death misclassification and under-reporting [40, 41], and only a few countries such as Ireland have robust systems for monitoring suicide attempts [37, 42, 43]. The WHO produced guidelines for prospective and standardized monitoring of suicide deaths and attempts to help countries act on the recommendation to improve surveillance [37, 44].

Prior to the release of the WHO report, the Government of Canada took some important steps to advance a national suicide prevention agenda. In 2012, the federal government passed Bill C-300, *An Act respecting a Federal Framework for Suicide Prevention* [10, 45]. Bill C-300 helped create a policy framework for suicide prevention which led to several initiatives by the Public Health Agency of Canada. One of the objectives of Bill C-300 was to increase public access to statistics [10]. To comply with the legislation and operationalize the WHO recommendations for improving data collection, PHAC developed the Canadian Suicide Surveillance Indicator Framework in 2017. The framework included suicide-related outcomes, and measures for risk factors including chronic pain and mental illness, and protective factors such as social support and sense of belonging [32, 46]. Outcomes included incidence rates of suicide mortality, hospitalization and emergency department visits for self-injury, and prevalence rates of suicide attempts and suicidal thoughts (Fig. 1a).

The inclusion of both fatal and non-fatal outcomes reflects the ‘iceberg’ model of suicide (Fig. 1b), which is a conceptual model for measuring the full extent of suicide-related outcomes in a population [23, 37, 47–49]. The iceberg model of suicide is based on three common patterns in population health. First, suicide deaths (the top level in the iceberg model; Fig. 1b) account for a very small percentage of all forms of fatal and non-fatal suicide-related behaviour in a population. Second, the least harmful outcomes such as suicidal thoughts occur most frequently. And third, many people who think about suicide do not seek help through the health system, and therefore cannot be identified with administrative or clinical data. Overall, the iceberg model helps illustrate the magnitude of suicide as a public health problem [47, 48].

### National health databases used in suicide surveillance

In this section, we describe the primary outcomes and corresponding datasets used in the Canadian Suicide Surveillance Indicator Framework (Fig. 1a and b). We examine the following five outcomes: suicide deaths, hospitalization due to self-injury, emergency department visits for self-injury, and self-reported suicide attempts and suicidal thoughts/ideation. The CSSIF includes additional outcomes and risk and protective factors [32] which we do not examine. We comment on how the data is derived for each primary outcome and identify potential limitations, providing the necessary background for an in-depth analysis of challenges related to Indigenous-specific surveillance.

#### Suicide deaths

Suicide deaths are recorded in a national dataset, the Canadian Vital Statistics Death Database (CVSD). In Canada, death registration is a federal legal requirement, and medico-legal investigations are the responsibility of provinces and territories [50, 51]. CVSD is an administrative dataset derived from an annual census of all provincial and territorial vital statistics registries [52]. The registries include demographic data and record the cause of death for all individuals who die in Canada each year. Deaths are coded by cause according to International Classification of Disease (ICD-10) system [46]. The data source for the CVSD includes death registration forms that contain information from funeral directors, and a medical certificate of cause of death from a physician or coroner.

For sudden, unexpected, and deaths under suspicious circumstances, legislation requires that a medical examiner or a coroner determine the final cause of death [53]. Medical examiners are physicians, whereas coroners are non-physicians in all jurisdictions except in Ontario. In most places, police or health care staff notify the coroner or medical examiner (C/ME) about a suspected case of suicide, homicide, and ‘accidental’ (unintentional) death. C/MEs then determine the final cause of death by performing autopsies and toxicological tests, reviewing medical and police records, and talking to families or other witnesses. Nationally, C/MEs investigate approximately 15% of deaths (approximately 35–45,000 deaths) each year [54]. In the provincial/territorial and municipal context, C/ME data is relatively accessible and comprehensive; Nova Scotia, New Brunswick, Nunavut, and Newfoundland and Labrador, and Toronto and Montreal use C/ME data for suicide surveillance, health system planning, research [12, 41, 51, 55–57]; other provinces/territories and municipalities likely have similar surveillance initiatives but may not publicly report data or results. National surveillance with C/ME data, by contrast, is not possible because of the lack of a national dataset, and the level of agreement between vital statistics and C/ME data on deaths coded as suicide is not known for all provinces and territories.

From 2006 to 2010, federal agencies maintained the Canadian Coroners and Medical Examiners Database (CCMED). The CCMED resembled a national dataset, though jurisdictions with medical examiners (Alberta, Manitoba, Nova Scotia, and Newfoundland and Labrador) did not contribute data [54]. CCMED was useful insofar as it contained data not covered in vital statistics and attempted to use standardized case and variable definitions [53]. Unfortunately, the data was not kept up-to-date, and the system was not expanded, which made it less useful, though this may change in the future. By virtue of the absence of national C/ME dataset, national public health surveillance of mortality is based on vital statistics.

#### Hospitalization due to self-injury

The second indicator is hospitalization due to self-injury. Following a suicide attempt, many individuals visit or are taken to the emergency department. Patients who have serious injuries or who are assessed as being at high-risk for self-harm may be voluntarily or involuntarily admitted to hospital for medical and/or psychiatric care. In Canada, the main indicator for this type of event is hospitalization due to intentional self-injury which includes intentional self-poisonings and self-inflicted injuries irrespective of suicidal intent [46, 58].

Hospitalization self-injury data comes from two sources of ‘hospital separations.’ A hospital separation is an episode of inpatient care that ends with discharge or death. The national data sources include the Discharge Abstract Database (DAD) and the Hospital Morbidity Database [46, 58]. The DAD includes mandatory reporting from all hospitals and health centres in the country except in Quebec [46, 58]. DAD uses ICD-10-CA codes to identify self-injury or self-poisoning in discharge diagnoses fields following hospitalization. Although the database reliably captures episodes of patient hospitalization, the major limitation is that DAD cannot document a patient’s intention to die [58], and therefore includes events related to both suicide attempts as well as non-suicidal self-injury, which is increasingly understood as a distinct outcome [31, 35]. For this reason, hospitalization is a proxy indicator for suicide attempts. A related limitation is that suicide-related behaviours may be under-captured in administrative data compared to clinical data because coding has poor sensitivity [59–61], which in turn underestimates the population burden of self harm.

In the context of rural and remote populations, indicators related to health service use such as hospitalization may differentially undercount suicide attempts. Many northern Indigenous communities do not have local access to a hospital; rather, they are served by nursing stations that provide the first point of health care in an emergency. Medically serious attempts that result in traumatic injuries or poisonings are usually transferred by flight to regional hospitals or southern tertiary care institutions and therefore would be counted in national and provincial hospitalization data. However, events with less severe injuries may not require hospitalization and may be managed and treated locally via telehealth [62].

#### Emergency department visits for self-injury

The third indicator in the suicide surveillance framework is the rate of emergency department (ED) visits for self-injury [32]. At present, Canada lacks a national dataset for emergency department care [63]. Since health care is primarily a provincial/territorial responsibility, much of the data from EDs is housed in clinical information systems such as electronic health records (EHRs). Studies from several provinces have shown that extracting ED data on suicidal behaviour from clinical databases is feasible [64–66]. However, provincial/territorial EHR systems are varied, and some do not use standardized codes for diagnoses, making it difficult to accurately and consistently capture cases of self-injury.

Instead of clinical data, the suicide surveillance framework uses data from the National Ambulatory Care Reporting System (NACRS). NACRS is an administrative database that compiles ambulatory care visit data for several provinces, territories, and health regions, and includes a set of demographic and clinical variables with ICD-10-CA diagnostic codes [67]. In 2015–2016, NACRS covered 64% of emergency departments across Canada, with complete coverage only for Alberta, Ontario, and Yukon, and no coverage for 3 provinces (Quebec, New Brunswick, and NL) and two territories (NU, NWT) [67]. Although NACRS is the largest ED care dataset, reporting is voluntary, and not nationally-representative.

The second ED data source used in the suicide surveillance indicator framework is the Canadian Hospital Injury Reporting and Prevention Program (CHIRPP). CHIRPP is a registry-based injury surveillance system that receives data from 17 participating health care facilities, most of which are urban paediatric hospitals [68]. Data collection involves patient and physician reporting forms completed during the ED visit. The recent creation of an electronic platform for CHIRPP improved the program’s timeliness, flexibility, and data management procedures [68]. However, CHIRPP has several limitations including varied case-capture rates, which range from 68 to 100% across reporting sites [68, 69]. Poor capture of injury cases at some sites may be due in part to the recording burden on patients and families during a distressing time. A recent study that compared CHIRPP to clinical records found that 27% of injury cases were not included in CHIRPP data, mostly because patients or parent/guardians did not submit the data collection form [69]. The study also found severe injuries were at increased risk of being missed by the registration system, as were cases of self-injury or self-poisoning [69]. Patient/parent participation might also be influenced by literacy, English and French language proficiency, and stigma [70, 71]. A subsequent CHIRPP study on self-injury found that the form captured complete details about injuries, but suggested that youth may under report self harm due to privacy concerns related to the data collection process [60]. Another limitation of CHIRPP is that population-based estimates of injury burden cannot be calculated because reporting hospitals do not have a complete capture of injuries for their catchment area, and the population at risk (the denominator in rate calculations) is unknown.

#### Suicide attempts and suicidal ideation

The fourth and fifth indicators in the suicide surveillance framework are self-reported suicide attempts and suicidal ideation (Fig. 1a and b). Clinical and administrative data includes populations with the most serious injuries who also have contact with the health system [31]. Measuring the incidence of suicide-related behaviour with routinely collected data is inherently limited because the majority of people with suicidal thoughts or attempts do not seek help from the health system and do not die by suicide [48, 49]. Health surveys are a more effective way to measure rates of non-fatal suicide-related outcomes at the population-level because they include people who do not have contact with the health system.

In Canada, several national and regional health surveys contain questions on self-reported suicidal ideation and attempts. The suicide surveillance indicator framework used data from the Canadian Community Health Survey (CCHS) to determine the prevalence of suicidal ideation and suicide attempts [32]. Since 2007, the CCHS has been conducted annually with a sample of approximately 65,000 people [72]. The survey asks participants: “have you ever [and in the past 12 months] seriously contemplated suicide?” and “have you ever [and in the past 12 months] seriously attempted suicide”[72]? These questions provide data for national and provincial/territorial estimates of lifetime and recent prevalence of suicidal ideation and suicide attempts. CCHS is nationally representative and contains a question about Aboriginal identity. However, by design the sample omits about 3% of the national population [72] including First Nations living on-reserve, military personnel, and institutionalized populations who are disproportionately made up of Indigenous peoples, such as prisoners [72, 73].

The baseline rates for all suicide-related outcomes in the CSSIF are benchmarks for three vital public health tasks: (1) tracking changes in rates over time; (2) evaluating population health interventions; and (3) assessing health equity. The third task is not yet part of the existing suicide surveillance framework [32]. Nonetheless, PHAC’s work is aligned with the WHO guidelines [31, 37] and is essential for suicide prevention in Canada.

### Epidemiology of suicide among Indigenous peoples in Canada

We have attempted to respond to the TRC’s Call to Action #19 by examining the most recent data for five suicide-related outcomes from the national suicide surveillance indicators framework. We collected incidence and prevalence data for Indigenous and non-Indigenous populations for a convenience sample of jurisdictions across four geographic scales: country (Canada), province (Alberta), territory (Nunavut), and health region (Northwestern Ontario). We captured the most recent statistics (as of May 2018) that were publicly available online from the following organizations: Statistics Canada [74–76], Public Health Agency of Canada [32], Canadian Institute for Health Information [77], Public Health Ontario [78], and Alberta Health [79], Nunavut Tunngavik Inc. [80], and the First Nations Information Governance Centre [81]. We compared incidence and prevalence rates between Indigenous and non-Indigenous or general populations. We report count and population data, crude and age standardized incidence and prevalence rates, and 95% confidence intervals (Tables 1 and 2); age-standardized rates were based on the 2011 Canadian standard population. We report rates for specific Indigenous groups including Inuit, First Nations, and Métis, and use nation- and region-specific terms where possible. Mortality and health service use data for Northern Ontario is for the North West Local Health Integration Network (NW-LIHN), which is an administrative division within Ontario’s health care system. Indigenous-specific outcomes based individual-level ethnic identifiers were not available for small areas in Ontario from open data sources. For mortality, hospitalization, and ED visit rates in Ontario, we used the NW-LIHN as a geographic proxy because it has the proportionately largest Indigenous population in Ontario compared to all other LIHN’s (~ 37%).

**Table 1:** Recent and lifetime prevalence of suicidal thoughts and suicide attempts among Indigenous populations in Canada

Region, Indigenous Group	Number of survey participants	Age Group	Suicidal Thoughts		Suicide Attempts		Source
			Recent	Lifetime	Recent	Lifetime	
Canada							
General Population	~ 65,000	15+	2.5%	12.3%	0.4%	3.4%	CCHS (2015)
First Nations (Off Reserve)	28,409^a^	18+	5.2%	14.7%	–	–	APS (2012)
Inuit	–	18+	5.5%	15.2%	–	–	APS (2012)
Métis	–	18+	3.8%	12.8%	–	–	APS (2012)
Nunavut							
Inuit	1,710	18+	14.0%	48.0%	5.0%	29.0%	IHS-NU (2008)
Inuit	1,581	18+	5.8%	16.3%	–	–	APS (2012)
Ontario							
First Nations (On Reserve)	1,500	18+	18.6%	25.3%	10.5% ^b^	13.4%	FNRHS (2008/10)
First Nations (Off Reserve)	4,286^a^	18+	6.4% ^b^	12.2%	–	–	APS (2012)
Métis	–	18+	3.2% ^b^	15.7%	–	–	APS (2012)
Alberta							
First Nations (On Reserve)	1,418	18+	15.2%	22.1%	–	14.4%	FNRHS (2008/10)
First Nations (Off Reserve)	3,765^a^	18+	5.5%^b^	18.6%	–	–	APS (2012)
Métis	–	18+	4.3%^b^	13.8%	–	–	APS (2012)

*CCHS* Canadian Community Health Survey, *APS* Aboriginal Peoples Survey, *IHS-NU* Inuit Health Survey-Nunavut. FNRHS=First Nations Regional Health Survey

^a^Total number of APS (2012) survey participants in region including all First Nation, Inuit, and Métis

^b^Interpret with caution; high sampling variability

**Table 2:** Incidence of emergency department visits for self injury, hospitalization for self injury, and suicide mortality among Indigenous and non-Indigenous populations in Canada

	Population (Year)	ED Visits for Self Injury				Hospitalization for Self Injury				Suicide Mortality			
		Cases, n=	CR	ASR	95% CI or SE	Cases, n=	CR	AR	95% CI or SE	Cases, n=	CR	ASR	95% CI or SE
Alberta													
First Nations	162,921 (2014)	1,373	838.99	716.01	(SE 29.4)	382	233.47	212.69	(SE 24.11)	73	44.81	45.59	(SE 23.21)
Non-First Nations	3,957,981 (2014)	5,763	142.9	142.96	(SE 1.91)	1,824	45.23	45.85	(SE 1.09)	454	11.47	11.8	(SE 0.57)
Ontario													
Northern Ontario	70,994 (2012)	449	632.9	615.6	(558.2–673.0)	131	184.7	183.7	(151.9–15.4)	28	39.4	39.1	(24.5–53.7)
Ontario, General Population	11,968,556 (2012)	15,976	129.5	132.4	(130.3–134.5)	7,620	61.8	63	(61.6–64.4)	1,223	10.2	10.2	(9.7–10.8)
Nunavut													
Inuit	30,424 (2014)	–	–	–	–	–	–	194	(146–242)	26	72.1	62.7	–
Canada, General Population	35,535,348 (2014)	–	–	–	–	–	–	66	(66–67)	4,254	12	12	–

*ED* Emergency Department, *CR* Crude Rate, *AR* Age-Adjusted Rate, *CI* 95% Confidence Interval, *SE* Standard Error; Not reported (−); Population estimates are not those used in the rate calculations; Nunavut Inuit population is from the Nunavut Beareau of Statistics; ED and hospitalization data is for 2015; mortality data is for 2012 in Ontario, and 2014 elsewhere; hospitalization and suicide rates for Nunavut are calculated based on the event and population for the territory including Inuit and non-Inuit. DATA SOURCES: Statistics Canada [74], Canadian Institute for Health Information [77], Public Health Ontario [78], and Alberta Health [79]

Overall, the suicide rate in Canada is similar to other high-income nations [31]. The rate declined slightly since the late 1970’s [46], and has been relatively stable during the twenty-first century [74]. In 2015, 4405 people died by suicide, and the age-standardized suicide incidence rate was 12.3 deaths per 100,000 population [74]. This made suicide the 9th leading cause of death overall [74], and the second leading cause among youth [46]. Based on the most recent data, our analysis revealed that across all five indicators, rates of suicidality were higher in Indigenous populations than in general or non-Indigenous populations.

Lifetime suicidal ideation (Table 1), compiled from a variety of data sources, ranged from 12.8% among Métis in Canada to 48% among Inuit in Nunavut, compared to 11.7% in the non-Indigenous population. Lifetime suicide attempt prevalence rates (Table 1) ranged from 13.4% among on-reserve First Nations populations in Ontario to 29% among Inuit in Nunavut; the general population rate in Canada was 3.4%. In Nunavut, estimates of the prevalence of suicidal ideation were three times higher (Table 1) in the Inuit Health Survey compared to the Aboriginal Peoples Survey (2012), which is a notable difference between sources. Incidence rates of emergency department visits for self-injury (Table 2) were 4 to 5 times higher in northern Ontario and among First Nations in Alberta compared to general population estimates. Rates of hospitalization due to intentional self-injury (Table 2) were 2.9 to 4.6 times higher in Indigenous populations than in general populations. Age-standardized suicide mortality rates (Table 2) were significantly higher among Indigenous compared to non-Indigenous populations, ranging from 39.1 deaths per 100,000 in northern Ontario to 62.7 deaths per 100,000 population in Nunavut.

A notable finding was the difference in the reported prevalence rates of suicidal ideation in Nunavut. The Inuit Health Survey and the Aboriginal Peoples Survey sampled a similar number of participants in the territory, with a similar response rate [80, 82], and both included questions about suicidal ideation. The difference in prevalence between the surveys may reflect changes in rates over time, as there was a five-year gap (2007/08 versus 2012) between surveys. The prevalence difference may also be related to the slightly higher proportion of women in the IHS-NU than in the APS (60% versus 55%) [75, 80], as women tend to have higher rates of self-reported SI. Methodological and governance differences in the surveys may also be a factor that influenced participant willingness to disclose sensitive health information.

Overall, publicly accessible statistics were not available for all indicators or geographic areas. For example, we were not able to find a public source of data on ED visit rates for Nunavut. A previous study with data from the Canadian Hospital Injury Reporting and Prevention Program identified 926 emergency department visits for intentional self-injuries over a 20 year period at data collection sites in Nunavut and Northwest Territories [83]. However, the study did not calculate incidence rates because the total number of people at risk was not known. A limitation of our own analysis is that we used the geographic proxy method to identify regions that were primarily Indigenous, such as Northern Ontario, and for select outcomes in Nunavut. We discuss the limitations of this approach in the next section. Notwithstanding these limitations, the most up-to-date data shows a clear trend: suicide continues to disproportionately impact Inuit and First Nations in Canada.

### Challenges in suicide surveillance among Indigenous populations

The Canadian Suicide Surveillance Indicator Framework draws data from national administrative databases and surveys. Although, such data sources provide good coverage of the population and high-quality information, the current data infrastructure in Canada has limitations that make it difficult to monitor suicide-related outcomes in Indigenous populations. In this section, we examine challenges in suicide surveillance related to outcome measurement, timeliness, geographic coverage, identification of Indigenous peoples, and data governance (Table 3).

**Table 3:** Overview of national health databases used in suicide surveillance

Outcome, Data Source, and Most Recent Year available	Definitions, Coding, and Information Sources	Geographic coverage	Population Exclusions	Indigenous identifiers	Other Challenges and Limitations
Suicide mortality Canadian Vital Statistics Database - Deaths (2016)	ICD-9 (pre 2000); ICD-10 (2000 - present). Death certificate from physician or funeral director. For ‘non-natural’ deaths, cause codes are determined by coroner/medical examiners	National	None	No	Under-reporting of suicide due to misclassification as unintentional injuries or undetermined intent; extent of misclassification may vary by jurisdiction.
Self-inflicted injuries, Hospitalization Hospital Morbidity Database (2011–2012) Discharge Abstract Database (2015–2016)	ICD-10-CA. Does not distinguish between self-injury with suicidal intent (suicide attempt) and without suicidal intent (non-suicidal self injury); lacks suicide-specific codes.	National except Quebec (DAD), National (HMDB)	Patients admitted to acute care psychiatric hospitals; Institutionalized populations;	No	Hospital separations include both discharges and deaths, therefore DAD has overlap with CVSD for suicide deaths that occurred during a hospitalization related to a suicide attempt; poor sensitivity in case coding contributes to undercounting.
Self-inflicted injuries, Emergency Department visit National Ambulatory Care Database (2015)	ICD-10-CA. Does not distinguish between self injury with suicidal intent (suicide attempt) and without suicidal intent (non-suicidal self injury), and lacks suicide-specific codes; Does not include data on visits related to suicidal ideation as these are not covered by ICD-10 codes.	64% of all EDs; Complete coverage for AB, ON, and YT; No coverage for 5 provinces/territories (QC, NL, NB, NWT, and NU)	None	No	Poor sensitivity in case coding contributes to undercounting and thus under-estimates of the population burden of self-harm.
Self-inflicted injuries, Emergency Department visit Canadian Hospital Injury Registration and Prevention Program (2016)	Registry-specific case definitions with narrative component; does not use ICD coding scheme. Patient/parent and physician completed forms, and medical record review	17 hospitals (primarily urban, paediatric facilities)	Children that present to general hospitals; Adults aged 18 and older; Rural populations including Inuit, First Nations, and Métis living on reserve or in rural or northern communities;	No	Under-coverage of events with higher injury severity such as trauma and suicide attempts; reporting burden on patients and clinicians; Literacy and English/French language requirements; Population-based rate estimates not possible because of unknown denominators due to a lack of defined catchment
Suicide attempts Canadian Community Health Survey (2015)	Standardized self-report questionnaire administered by telephone. Survey question: “Have you ever [and/or in the past 12 months] seriously attempted suicide?”	National sample, but elevated non-response rates in territories and rural regions	Excludes populations that tend to report higher rates of suicidal ideation and attempts: First Nations living on-reserve, military personnel, and institutionalized populations such as people in prison, hospital, or foster care.	Yes	n/a
Suicidal thoughts Canadian Community Health Survey (2015)	Standardized self-report questionnaire administered by telephone. Survey question: “Have you ever [and/or in the past 12 months] seriously contemplated suicide?”	National sample, but elevated non-response rates in territories and rural regions	Excludes populations that tend to report higher rates of suicidal ideation and attempts: First Nations living on-reserve, military personnel, and institutionalized populations such as people in prison, hospital, or foster care.	Yes	n/a

#### Conceptualizing and measuring suicide

One of the major challenges in suicide surveillance and research is the lack of a shared set of definitions of suicide and non-fatal outcomes. Public health and clinical disciplines have not reached consensus on a nomenclature for the spectrum of suicide-related thoughts, communications, behaviours, and consequences [31, 84, 85]. In part, definitions are elusive because suicide is not a disease with a singular or observable cause. Rather, suicidality is described as an event or a psychological state with intersecting and compounding risks that can emerge over a lifetime [35].

One of the tasks in conceptualizing and measuring suicide is determining the intent leading up to an act of self-harm. For example, it can be difficult to tell the difference between an overdose that was on purpose or accidental, or to distinguish between suicidal and non-suicidal (self) cutting. Making these distinctions is challenging for clinicians; it is also difficult in retrospective research using secondary data. As a result of the ambiguity, population health assessments often use broad categories of self-harm as proxies for suicide deaths and attempts.

ICD-10 codes for “intentional self-harm” are used to classify suicide deaths and attempts; in Canada, ICD-10-CA is used for hospitalization data. ICD codes cannot distinguish between intentional self-injury with or without the desire to die (suicide attempt versus non-suicidal self-injury) [35], nor do the codes capture episodes of suicidal thoughts in the absence of self-injurious behaviours. Recent studies of emergency department visits used “suicide-related behaviour” and “self-harm” with inclusive definitions that captured non-fatal suicide attempts due to self-poisoning or self-injury, along with events with undetermined intent [65, 86]. Inclusive definitions are used by national statistical agencies because ICD codes under-estimate suicide attempts by more than 50% [61, 66, 87].

Historically, evidence has shown that vital statistics data may under-report and misclassify suicide deaths as unintentional or undetermined injuries, which can contribute to underestimated suicide rates [39–41, 88–90]. Yet, a 2017 study found high rates of concordance (up to 98.8%) between C/ME and vital statistics data in Ontario [91], which suggests that misclassification may be less of a problem in some provinces and territories. However, agreement between C/ME data and vital statistics likely varies across jurisdictions. Overall, the lack of universal definitions for outcomes makes it difficult to accurately measure the burden in a population or to compare rates between two populations.

#### Low base rates, ‘rare events,’ and small populations

One of the universal challenges in assessing the impact of public health interventions on suicide is the low base rate [92]. Even though rates may be especially high in some northern Inuit and First Nations communities, the actual number of deaths is low compared to cities in southern Canada. Suicide is considered a ‘rare event,’ therefore it is difficult to determine whether a change in the absolute number of cases is attributable to an intervention [92]. In a northern and rural context, this is made even more challenging because it is difficult to detect statistically significant changes in small populations such as those in the Arctic. This is also challenging because public health approaches to suicide prevention are multifaceted; it is hard to discern which intervention components contribute to changes in mortality rates.

#### Timeliness of data access

Timely data access is another challenge is suicide surveillance. Administrative data sources often have lengthy delays between event occurrence and data release. In May 2018, the most recent national mortality data available were for 2015 [74]; data for non-fatal indicators are updated faster, usually in two years or less. There are likely several reasons for these time lags. Health information systems are complex; they require prompt and standardized submissions from multiple jurisdictions. Although Canada has universal health care, in reality, care is not delivered by a single system, but rather thirteen provincial/territorial healthcare systems, and one federal system for specific groups including federal prisoners, veterans, and on-reserve First Nations. The two or more year time lag before administrative and other secondary data can be used makes it difficult for public health systems to identify and be responsive to trends.

As an alternative to vital statistics data for monitoring deaths, some provinces/territories and municipalities use coroner and medical examiner data because it can be more timely. In Newfoundland and Labrador for example, the provincial health statistics agency conducts an annual census of suicide from C/ME cases, and maintains a database that can be used for surveillance and research [41]. In addition to being more current, C/ME data also has the advantage of capturing more detailed information about method and precipitating factors than vital statistics, though there is variation in the amount and quality information collected by C/MEs, and mental health history is inconsistently recorded [51, 56]. A key limitation in using C/ME data for national surveillance is that to get a complete dataset with all suicide deaths, information would have to be extracted from the 13 chief C/ME offices across the country [54]. Data extraction from each C/ME office would require substantial resources since C/ME data is often recorded on paper rather than in digital form in some jurisdictions. Statistics Canada has developed a data capture tool to improve the consistency of data entry and transmission, which is being used by several provinces and territories [54]. However, data processing may still be time consuming. Relatedly, there is no national system of electronic clinical records to capture non-fatal behaviours such as suicide attempts and suicidal ideation.

#### Geographic coverage

Incomplete geographic coverage is another challenge for suicide surveillance. Of the five databases used in the PHAC suicide surveillance indicator framework, only vital statistics has complete national coverage and three databases exclude one or more provinces or territories (Table 3). CHIRPP has limited coverage outside of urban centres, especially in rural and northern regions where Indigenous peoples make up most of the population. NACRS also has limited or no coverage in more rural provinces such as in Atlantic Canada, and in territories and health regions that are predominantly Indigenous. More than half of Indigenous people in Canada live in cities where NACRS coverage is good, but the absence of Indigenous identifiers in health data is a barrier to producing estimates for provinces with large urban Indigenous populations. The CCHS excludes participants who live on reserves and in communities in the territories. Overall, rural regions in Canada face undercoverage in several administrative and survey datasets.

#### Indigenous identifiers

Another challenge in suicide surveillance is the lack of ethnic identifiers [18]. According to the 2016 census, there are 1.6 million Indigenous people in Canada [93]. In broad terms, the population is comprised of three ethno-cultural groups: Inuit, First Nations, and Métis. Within these constitutionally defined groups, there is immense diversity in culture, language, traditional territory, political self-determination, colonial history, and social, economic, and health status. Provincial and territorial governments differ in how they approach identifying Indigenous people in health information systems: some jurisdictions include Indigenous identifiers, and some do not. In Nunavut, where Inuit comprise the majority of the territory’s population, healthcare card numbers include a digit that identifies individuals as Inuit. By contrast, in Newfoundland and Labrador where Inuit and First Nations are proportionately small populations, healthcare card numbers do not specify ethnicity, nor do other provincial databases. In British Columbia, provincial vital statistics are linked with health insurance and other registries with ‘Aboriginal status’ identifiers [94]. The lack of a standard and universal method for ethnic identification across provincial, territorial, and federal health systems makes it difficult to produce comparative or national statistics on Indigenous populations.

Within the CSSIF, four of the five databases do not include broad or specific Indigenous identifiers. Only CCHS identifies Indigenous survey participants as Inuit, First Nation, or Métis based on self-reported identity. High-income countries with large Indigenous populations such as Australia, New Zealand, and the United States include Indigenous identifiers in vital statistics, administrative, and survey data [18, 21]. However, circumpolar countries like Norway and Finland do not [95]. In Canada, the omission of ethnic identifiers in some administrative datasets makes it difficult for communities, governments, and researchers to compare rates of suicide for specific ethnic groups with the general population [18].

One of the ways that researchers and governments in Canada cope with the absence of Indigenous identifiers is to link administrative data with government registries such as the Indian Register or Non-Insured Health Benefits lists [18]. However, this method can miss individuals who are not registered under these programs, such as Métis, non-status First Nations, and some Inuit [18]. In 2014/15, the NIHB program included 779,300 First Nations and 44,733 Inuit [96], but did not cover nearly 40% of the self-identified Indigenous population in Canada. One of the reasons for this was that specific Indigenous peoples do not qualify for health benefits under the federal government program. A second reason is that select jurisdictions such as British Columbia and Nunatsiavut, the Inuit region in northern Labrador, directly administer non-insured benefits rather than going through Health Canada, and therefore maintain a separate client list [96]. Another data linkage option is to use the census. The Canadian census includes an “Aboriginal identity” question, which provides a comprehensive capture of people who self-identify as Indigenous. Although these two methods are feasible, routine and timely data linkage for surveillance and research on suicide among Indigenous populations has not occurred.

An alternative and commonly used approach for measuring mortality among Indigenous populations is the “geozone” method [4, 7, 18]. The geozone method involves using census data to identify geographic areas where a majority of the population self identifies as Inuit, First Nations, or Métis. This is an ecological approach that is most pragmatic in rural and northern regions where communities are primarily Indigenous, such as on reserves or in the Arctic. However, there is a risk of the ecological fallacy with this method and it may underestimate health disparities [18]. Overall, area-based approaches are less useful for cities in southern Canada and the increasingly urbanized and culturally diverse regional centres in the North.

Simply put, governments do not know how many Indigenous people die by suicide each year in Canada, nor where the burden is concentrated. Nor is it known how many Indigenous people visit the emergency department or are hospitalized after attempting suicide. The current approaches to Indigenous identification in health data are varied, haphazard, and have several threats to quality. The lack of a standardized and comprehensive approach to Indigenous identification in health data camouflages inequity [18] and impedes outcome monitoring in suicide prevention. Resolving this challenge likely requires technical, legislative, and political support.

#### Indigenous data governance

A final and substantial challenge in suicide surveillance is the absence of Indigenous governance over data. This absence is problematic because health statistics often construct and perpetuate stigmatizing narratives of illness in Indigenous health [97]. Historical and contemporary research by non-Indigenous scholars and institutions has tended to reinforce dominant cultural discourses of indigeneity as pathology, and characterize Indigenous communities as “desperate, disorganized, and depressed environments”[98] (p.34). Such depictions are compounded by deficit-focused studies that often fail to measure strengths and assets in Indigenous communities [18, 21, 99]. Even though ethical standards and methodological frameworks for Indigenous health research are well-established in federal research policies, academic institutions, and community settings in Canada, problematic research practices persist [100–103]. Similarly, public health surveillance by government agencies can pose similar risks if Indigenous communities are excluded from decision making about the process and control of the data [97].

Indigenous scholars and leaders have raised important critiques about epidemiological studies that neglect to meaningfully engage Indigenous communities as rights-holders, collaborate with communities implicated in data, misinterpret results, focus on descriptions of problems rather than interventions, and fail to adhere to ethical guidelines for Indigenous research [18, 99, 104–106]. Indigenous communities, organizations, and governments also appear to have, at best, a limited role in overseeing national health information systems [18, 19]. For example, the Vital Statistics Council of Canada which provides oversight and operational direction for the collection of data on births and deaths [52], has representation from provinces and territories as well as Statistics Canada; however, Indigenous representation is notably absent from the council. Similarly, Indigenous organizations have been absent from some key national dialogues on equity in health status and health system performance measurement [107].

The lack of Indigenous involvement in national health information governance is problematic because there is an ethical imperative to be inclusive. Indigenous peoples have the right to sovereignty over resources and decisions that impact wellbeing and Indigenous control over health information is an essential aspect of community empowerment and self-determination [18, 21]. Health data is a crucial resource, and data-informed decision-making can help drive change in Indigenous health and suicide prevention [17, 21, 108]. As such, Indigenous communities, organizations, and scientific networks are asserting sovereignty over research, and increasingly over health data [18, 21]. Canada and other high-income countries such as Australia, New Zealand, and the US are without a national policy framework for Indigenous data governance [21], though in Canada national initiatives have emerged for the governance of survey data. The First Nations Regional Health Survey and the Inuit Health Survey are two examples. The surveys were designed by First Nations and Inuit partners to reflect priorities, and were rooted in the values and research principles specific to Indigenous governance. Of critical importance for First Nations populations, the sampling frame for the FNRHS is for on-reserve First Nations, who are otherwise excluded from the APS and other national surveys. Along with localized initiatives [26, 109, 110], these examples illustrate the fundamental shift in the assertion of rights over Indigenous population health data and how it is governed. They also provide instructive models for suicide surveillance.

### Improving suicide surveillance to support suicide prevention in urban and rural Indigenous communities

Health systems are unable to determine the impact of interventions without the ability to tease out rate differences between population groups. As a result, governments may over-invest in interventions that have limited or negative effects on outcomes for the highest risk populations or under-invest in services that work because gains went unmeasured. In the US, Indigenous communities in Alaska and Arizona developed suicide surveillance systems that were locally controlled and integrated with intervention planning [111, 112]. These systems used clinical, administrative, and registry data to support outreach and follow-up care with community members, and broader public health intervention planning. Surveillance data from the Arizona setting helped provide evidence that a regional prevention strategy contributed to a reduction in suicide deaths and attempts among Indigenous youth [112].

Notwithstanding the value of local surveillance, Canada needs a national suicide surveillance system that provides equitable coverage of Indigenous populations and communities, and can inform suicide prevention policy with systematically collected data. In this section, we propose strategies to strengthen the existing Canadian Suicide Surveillance Indicator Framework by enhancing capacity for suicide surveillance among rural and urban Indigenous populations. Our recommendations aim to build on the positive developments in suicide surveillance and the strengths of Canada’s existing administrative and survey data. Our goal is to help stimulate interest in creating a comprehensive and equity-focused suicide monitoring system that is useable by stakeholders in all contexts, from community-based organizations to federal government departments.

A first step to enhance the national suicide surveillance system is to develop a collaborative and inclusive governance model that recognizes the stake Indigenous communities and other socially excluded populations have in suicide prevention. This step would align with the WHO recommendation to create a “permanent task force that is specifically responsible for monitoring and improving the quality of suicide-related data” [31] (p. 102). Increasingly, Indigenous health systems and research are being transformed and redesigned to reflect the values and ways of knowing that are specific to Indigenous communities [22, 113–115]. As an extension of these changes, efforts to build a public health surveillance system that is relevant to Indigenous peoples must reflect the diverse interests and perspectives of the communities who are represented in the data and those who use it [22, 109]. However, decisions about the governance of data cannot assume a uniform perspective – Inuit, First Nations, and Métis communities, organizations, and scholars in northern, rural, and urban contexts may have distinct values, concerns, and interests with respect to how suicide is monitored. So too may other stakeholder groups that face differential impacts from suicide such as gender non-binary and sexual minority communities. Part of an inclusive approach to governance should involve establishing an independent community and scientific governing council. In the United Kingdom, the National Confidential Inquiry includes a Independent Advisory Group comprised of researchers and members of the public. In the Canadian context, such a council should include representatives from communities that face differential impacts from suicide, and should publicly report on membership and activities.

A second step to improve the national suicide surveillance system is to add “equity stratifiers” [116] to all data sources, including Indigenous identifiers. Health systems can work to integrate and validate Indigenous identifiers by routinely linking databases with suicide-related outcomes to databases that contain ethnic identifiers, including self-reported sources such as the census, and registry-based systems such as the Indian registry and non-insured health beneficiary lists. Using multiple sources would provide both conservative and inclusive rate estimates based on the varied approaches to identifying Indigenous people at the record-level. In Nunavut, this is already done with coroner data, which is publicly reported on an annual basis and stratified by region, age group, gender, and ethnicity.

A third step is to increase geographic coverage of administrative and health survey data to make them truly national in scope. A national data system will require mandating and standardizing administrative or clinical health information from emergency departments, conducting an annual census of C/ME records, and harmonizing federal and Indigenous health surveys such as the First Nations Regional Health Survey and the Inuit Health Survey. An interim or alternative approach to capture high-quality and longitudinal data based on health system visits could include setting up monitoring systems in representative locations [31] including urban, rural, and northern sites. A multi-site based monitoring initiative would have the advantage of being able to contextualize local data in knowledge about a specific place and inform interventions that are designed and led by the community. The second advantage would be that data for multiple sites could be combined to inform a broader understanding about the distribution of suicide-related contacts with the health system.

The Multicentre Study of Self Harm in England is an example of distributed, site-based system that collects detailed information about patients who are treated in hospital following self harm. A recent analysis showed that routinely collected hospital data undercounted self-harm events and underestimated incidence compared to the Multicentre Study data [117]. Such limitations need to be considered when using Canadian administrative data [59, 60], as might combining multiple sources of information [60]. Relatedly, a factor that needs to be considered for future surveys is content duplication. The health component of the Aboriginal Peoples Survey overlaps in several domains with the Inuit Health Survey, including with questions about suicidal ideation, though, the IHS includes additional questions about suicide attempts. Research fatigue is a reality in small and often-studied populations – this is the case for many communities in the Arctic. Minimizing redundancy in research and being minimally intrusive at the community level by using existing data sources rather than replicating is a necessary consideration for suicide surveillance and for population health research.

A fourth step is to improve the quality, comprehensiveness, and timeliness of suicide data. This step should include efforts to create a standardized medico-legal investigation framework and seek consensus on suicide-related outcome definitions and measures. This step could also involve integrating additional data sources into suicide surveillance including data from medical charts, EHRs, and police records, and exploring opportunities for using technological innovations to create real-time monitoring applications to detect suicide clusters and identify emerging at-risk populations. Such innovations should accompany efforts to improve data quality overall, not only for Indigenous populations. Efforts to enhance suicide surveillance should also be inclusive and support intersectional analyses. Initiatives should improve data quality and coverage for other minority groups, and embed or link equity stratifiers such as age, sex, non-binary gender, income, education and geographic location, in addition to ethnicity, sexual orientation, disability, and immigration status in all datasets [107, 116].

A fifth step is to create a harmonized suicide surveillance system that is accessible to Indigenous and local governments, frontline, clinical, and public health staff, community organizations, and health system decision-makers. At the regional level, data-informed decision making needs to be directed by stakeholders with contextualized knowledge, while also respecting the need for privacy and confidentiality related to data collection in small communities. Community leaders and organizations, Indigenous governments, and local clinicians are well positioned to understand community assets and priorities, design contextualized programs and policies, and use evidence from public health surveillance to deliver interventions where and when they are needed most. This would be facilitated by a mechanism for disaggregating national data into small areas to improve evidence of local variability and focus interventions on regions where rates are highest and on populations with emerging risks [7, 27, 38, 99]. Better data access will also support efforts to evaluate local interventions [118]. Large-scale federal and provincial/territorial surveillance initiatives would be complemented by government investments in community-based population health monitoring that covers suicide and mental health-related outcomes, as well as risk and protective factors.

## Conclusion

In 2009, the Truth and Reconciliation Commission was established to investigate the experiences and impact of the residential school system on Indigenous peoples in Canada. Residential schools were part of a sweep of colonial policies whose express purpose was the assimilation and enculturation of Inuit, First Nation, and Métis into white, settler, Euro-Canadian society. The TRC’s work made visible the direct link between colonization, intergenerational trauma, and the persistent health disparities experienced by Indigenous people. Suicide is one of the sharpest markers of this reality. Yet, this is a marker that can, in some ways, be difficult to see in official statistics.

Health systems that prioritize health equity must take steps to detect variations in health status and compare differences between general populations and those at the margins, at risk, or who are otherwise invisible in statistics. Stakeholders can work together to respond to the TRC by establishing a shared framework for governing national health data to track progress towards better health. Improving the quality of suicide surveillance and Indigenous health status monitoring can be part of the process of reconciliation in public health.

A comprehensive public health approach to suicide prevention in Indigenous communities requires more than simply gathering better data – it requires social change. The path to social change must be rooted in an understanding that the origins of suicide risk for Indigenous peoples were intentional and socially engineered. Many Indigenous communities face concentrated and intersecting vulnerabilities for suicide due to social exclusion, economic inequality, and systemic discrimination. Redressing such circumstances requires suicide prevention to extend beyond the borders of healthcare. Suicide prevention in Indigenous communities must be founded on a broader effort to reinstate Indigenous knowledge and sovereignty over resources and services, enshrine human rights in public policy, take steps to improve social equity, and promote health across the life course [8, 9, 11]. There also must be additional efforts to expand the knowledge base [99, 118]. While better data on its own does not prevent suicide, improving suicide surveillance can help track progress towards health equity and help keep governments accountable for funding evidence-based and community-designed interventions to prevent suicide.

## Abbreviations

C/ME: Coroner and medical examiner
CCHS: Canadian Community Health Survey
CCMED: Canadian Coroners and Medical Examiners Database
CHIRPP: Canadian Hospital Injury Reporting and Prevention Program
CSSIF: Canadian Suicide Surveillance Indicator Framework
CVSDD: Canadian Vital Statistics Death Database
DAD: Discharge Abstract Database
ED: Emergency department
EHR: Electronic health record
ICD: International classification of diseases
NACRS: National Ambulatory Care Reporting System
NAYSPS: National Aboriginal Youth Suicide Prevention Strategy
NIHB: Non-Insured Health Benefits
NL: Newfoundland and Labrador
NU: Nunavut
NW-LIHN: North West Local Health Integration Network
NWT: Northwest Territories
PHAC: Public Health Agency of Canada
TRC: Truth and Reconciliation Commission
WHO: World Health Organization
